# Functional Outcome in Bipolar Disorder: The Big Picture

**DOI:** 10.1155/2012/949248

**Published:** 2011-09-27

**Authors:** Boaz Levy, Emily Manove

**Affiliations:** Mental Health Counseling, Department of Counseling and School Psychology, University of Massachusetts, Boston, MA 02125, USA

## Abstract

Previous research on functional outcome in bipolar disorder (BD) has uncovered various factors that exacerbate psychosocial disability over the course of illness, including genetics, illness severity, stress, anxiety, and cognitive impairment. This paper presents an integrated view of these findings that accounts for the precipitous decline in psychosocial functioning after illness onset. The proposed model highlights a number of reciprocal pathways among previously studied factors that trap people in a powerful cycle of ailing forces. The paper discusses implications to patient care as well as the larger social changes required for shifting the functional trajectory of people with BD from psychosocial decline to growth.

## 1. Introduction

Psychosocial functioning in bipolar disorder (BD) runs the full gamut of human potential. Whereas some people with BD accomplish historical landmarks in human achievement [1–3], others experience significant difficulties in managing tasks of daily living [4]. The remarkable functional variability in BD highlights an inherent prognostic complexity [5–7], which is not immediately evident in the diagnosis [8]. Many studies have illuminated various aspects of illness progression in BD [9–11], yet significant improvement to functional outcome may require further theoretical and clinical advancement [12]. 

The astounding functional differences among people with BD present one of the toughest challenges to this effort, as these emerge across the entire spectrum of human development [7, 13–16]. Early emotional abnormalities and poor premorbid functioning tend to occur in BD [17–19]; however, adequate psychosocial adjustment prior to the first manic episode is also common [20–22]. Furthermore, after illness onset, many people with BD regain psychosocial functioning [13, 23], yet others suffer inordinate functional decline, which progresses from a state of psychosocial adjustment to a state of disability [23, 24]. The latter group is of particular interest to clinical research. Understanding the nature of the sometimes dysfunctional trajectory of BD may help to diminish it, and thereby reduce suffering and cost. 

This paper examines the strongest predictors of functional outcome in BD, which have been separately summarized in multiple previous reviews. In this regard, the paper does not aim to provide a comprehensive review of studies linking each of the factors under discussion to psychosocial functioning in BD. Instead, it offers an integrated view on previously reviewed findings and discusses potential implications for prevention and patient care.

## 2. Direct Effects of Cognitive Dysfunction

Cognitive impairment is among the strongest predictors of psychosocial disability in BD [6, 25]. Cross-sectional studies, now summarized in several comprehensive reviews and meta-analyses, indicate that cognitive deficits often persist into periods of euthymia [26–28], especially in people who suffer from marked psychosocial impairment during affective remission [29, 30]. The co-occurrence of cognitive and psychosocial impairment in the absence of mood symptoms advanced hypotheses about the ill effects of cognitive dysfunction on psychosocial adjustment in BD [31, 32]. Although practical considerations limit investigative efforts to nonexperimental evidence, this hypothesis gained considerable support from longitudinal studies that employed cognitive measures to predict long-term functional outcome in BD [13, 25, 33–37]. Longitudinal predictions that account for the confounding effects of mood symptoms suggest that cognitive impairment diminishes psychosocial functioning in BD [13, 25, 34]. 

In a broad view, the logic behind linking cognitive impairment to psychosocial disability in BD may parallel the reasoning that has created this association in dementia. The resemblance between cognitive symptoms of BD and those of dementia often goes unnoticed, because the degree of functional limitation can differ substantially between these disorders. Whereas the large contribution of cognitive impairment to psychosocial decline is widely recognized in dementia, the effects of cognitive impairment on functional outcome in BD may be more selective and subtle. Relative to that in dementia, the cognitive impairment in BD is milder, and the disruption to psychosocial functioning is less dramatic; however, the basic notion linking cognitive impairment to psychosocial disability is similar in nature. In some respects, people with BD who suffer from significant functional disability during euthymia may experience the illness as an attenuated or subclinical form of dementia. This view is supported by evidence that cognitive impairment in BD tends to be progressive over the course of illness [38–40] and correlates with psychosocial decline [41]. 

Cognitive impairment is milder in BD than in certain forms of dementia (e.g., Alzheimer's type) partly because it is not characterized by a severe amnesic syndrome [42]. The core dysfunction in BD during euthymia is executive in nature [43, 44]. Some researchers have even suggested that deficits in learning and memory are probably secondary to executive impairment [45]. The absence of a severe amnesic syndrome spares basic learning capacities and psychosocial functions; however, the executive dysfunction in BD may be significant enough to quickly limit the utility of preserved functions, particularly as task complexity increases [7, 32]. In BD [46] and in geriatric populations generally [47–49], disturbances in executive functioning have been tied to difficulties in accomplishing tasks of daily living. Studies also indicate that executive dysfunction in BD predicts poorer academic performance [50], worse vocational outcomes [25, 51], reduced social adjustment [52], and diminished quality of life [53, 54]. 

Thus, although cognitive impairment in BD is not completely incapacitating, the balance of the data suggests that it generates significant disruption to social and vocational adjustment [6, 13, 55, 56]. In other words, people with BD who suffer significant executive impairment during euthymia may not need custodial care, but they probably struggle to fit into mainstream environments, where functional expectations are typically set for the cognitively intact.

## 3. Direct Effects of Illness Severity

Illness severity is another strong predictor of psychosocial disability in BD [57]. Younger age of onset [58], longer duration of mood episodes [6], higher number of psychiatric hospitalizations [51], lingering residual symptoms [59, 60], psychosis [61], and substance use disorders [62, 63] all predict greater psychosocial dysfunction in BD.

The argument for a direct impact of illness severity on psychosocial functioning in BD probably provides the most intuitively appealing explanation for the correlation between these two variables. Younger age of onset disrupts psychosocial development at an earlier stage, altering the trajectory of educational, professional, and interpersonal growth [64, 65]. In addition, the break of psychiatric illness early in life likely carries deleterious effects on identity development [66]. Coupled with the stigma associated with mental illness generally and BD in particular [66–71], these internal effects may hamper efforts to achieve social adjustment [69, 71]. Further challenge to these efforts comes from recurrent mood episodes and frequent hospitalizations over the course of illness, imposing inconsistency to educational and vocational pursuits and repeated disruption to interpersonal engagement [5, 66, 68, 72–74]. Lingering residual symptoms between mood episodes impede efforts to reengage with psychosocial demand [25, 30, 33], and thereby make functional recovery after hospital discharge more challenging [75]. Finally, episodes of psychosis and chronic substance misuse contribute to an erratic course of development [61, 62]. The emotional and behavioral lack of control associated with substance use and psychosis diminishes the likelihood of obtaining psychosocial adjustment later in life [61, 62]. Taken together, all of these factors carry direct effects on psychosocial functioning and development in BD.

## 4. The Link between Cognitive Dysfunction and Illness Severity

The direct impact of cognitive impairment and illness severity on psychosocial functioning in BD may be compounded by the potential synergy between these factors. An increasing volume of studies indicate a robust association between illness severity and cognitive functioning in BD [40, 76, 77]. In particular, the number of mood episodes negatively correlates with cognitive functioning in a number of domains, including executive functioning and verbal memory [78]. In addition, cognitive dysfunction in BD is associated with the number of hospitalizations and the duration of mood episodes [77, 79]. These studies advanced the hypothesis that a more severe course of illness leads to progressive cognitive decline in BD in a process that may involve neurodegeneration [76, 80]. 

The neurodegenerative hypothesis holds that chronic mood instability generates physiological stress with neurotoxic effects, leading to neurological damage and cognitive decline over the course of illness [76, 80]. Within this model, Kapczinski et al. [80] applied the notion of “allostatic load” (AL) to BD. AL generally refers to the “wear and tear” of biological systems that occurs during physiological adjustment to stress, whereby this process becomes distorted and no longer efficient. In biomedicine, the concept of AL captures the biological toll of adaptation to excessive stress [81]. The higher rates of morbidity and mortality found in people with BD due to medical conditions not directly related to their psychiatric disorder, such as cardiovascular disease, obesity, diabetes mellitus, and metabolic syndrome [82–84], evince the deleterious physiological effects of stress in BD [85, 86]. Evidence for possible effects of stress on the brain comes from neuroimaging studies that found morphological abnormalities in BD [87]. In a recent review, Arnone et al. [87] concluded that BD is associated with whole brain and prefrontal lobe volume reductions, along with volume increases of the lateral ventricles. There is evidence that these and related brain abnormalities in BD are associated with both cognitive [88] and psychosocial decline [89]. Taken together, these studies suggest a stress-related cognitive, neurological, and psychosocial decline in people with BD who suffer from a more severe course of illness. 

From a broader perspective, the link itself—between illness severity and neurocognitive decline—may aggravate the direct effects that each of these factors have on psychosocial functioning in BD. Thus, a more severe course of illness reduces psychosocial functioning in BD and simultaneously decreases neurocognitive functioning, which then also directly lowers functional outcomes. Thus, the two factors that have the strongest direct effect on psychosocial functioning in BD may be looped together in a way that accelerates functional decline.

## 5. Anxiety

BD has a particularly high rate of comorbid anxiety disorders estimated at over 50% in several studies [90, 91]. Intense anxiety in BD predicts a more severe course of illness and poor prognosis [92, 93]. A number of studies found that people with BD who suffer chronic anxiety tend to have a younger age of onset, longer and more frequent mood episodes [94, 95], higher prevalence of substance use disorders [96], decreased response to lithium and anticonvulsant medication [92, 94, 97], and increased suicidal ideation and attempts [98]. Coupled with illness severity, comorbid anxiety disorders strengthen the prediction of poor functional outcome in BD, as indicated by lower GAF scores, decreased social role functioning, poorer quality of life, and minimal employment [95, 99, 100]. 

Anxiety, which may reflect a natural emotional reaction to the instability that inheres in severe psychiatric disorders, may also exacerbate illness severity and functional deterioration through relatively underinvestigated pathways. One such pathway may involve the potentially negative impact of anxiety on cognitive functioning [101]. High levels of anxiety can significantly compromise attentional control and decision making even in nonpsychiatric populations [102, 103]. Well-designed studies indicate that neuropsychological test scores, across 6 cognitive domains, tend to be particularly sensitive to hypothalamic-pituitary-adrenal (HPA) axis dysregulation and elevated levels of cortisol [103, 104]. The HPA axis in BD can be dysregulated across all clinical states, including euthymia [105, 106], and may affect cognitive functioning. Thus, HPA axis dysregulation can lead to debilitating cognitive impairment not only through the neurotoxic effects of inordinate allostatic loads, but more directly through excessive sympathetic arousal, triggered by the cognitive challenges of daily living. In short, it seems feasible to hypothesize that acute anxiety may compromise cognition in BD. Anxiety can potentially make baseline cognitive impairment circumstantially more acute and thereby further decrease functional abilities. 

Another pathway in which anxiety may compromise psychosocial function in BD could be related more specifically to the encounter between cognitive impairment and demand in psychosocial contexts. This encounter may produce anxiety, especially when the person is unable to meet expectations in highly visible social circumstances. Thus, while anxiety compromises cognition, cognitive challenges in a cognitively compromised state can trigger anxiety. Even in nonpsychiatric populations, cognitive challenges significantly increase anxiety and physiological arousal [101]. Physiological anxiety in nonpsychiatric subjects increases during cognitive testing and rises even further when subjects make errors [107]. These effects are more intense in people who suffer from mental illness or substance use disorders, even during remission or abstinence [108, 109].

Since the most common behavioral reaction to anxiety is avoidance [110], people with BD who experience cognitive impairment may tend to withdraw from psychosocial demands that evoke anxiety to decrease their experiences of social failure. More broadly, the encounter between cognitive impairment and demand in daily life can create anxiety that exacerbates cognitive deficits, limits functional ability, reduces motivation, and leads to avoidance of psychosocial engagement. In schizophrenia research, several studies suggest that an avoidant coping style mediates the link between neurocognitive impairment and psychosocial functioning [111, 112]. Although there is little direct evidence that psychosocial avoidance plays a similar role in people with BD, this hypothesis remains viable, given the similarities between cognitive impairment in BD and schizophrenia [111, 113]. In summary, the interplay between anxiety and cognitive impairment may further limit functional capacities and exacerbate psychosocial decline in BD. 

## 6. Diathesis-Stress

Various diathesis-stress [86, 114] and related models [115, 116] in BD research highlight the interactions between genetics and environmental stress as important predictors of illness onset and severity. These models broadly hold that cumulative environmental stressors trigger a person's genetic predisposition to experience mood disturbance and affect the progression of the illness after onset [117, 118]. In a recent review, Bender and Alloy [114] examined evidence for three of these models—the kindling hypothesis of illness progression in BD [119], the behavioral approach system (BAS) dysregulation model [120], and the social rhythm disruption (SRD) model [121]. 

The kindling hypothesis asserts that major stressful life events (SLEs) are required to trigger initial episodes in BD, but then, subsequent episodes become progressively uncoupled from stressors, to the point that future episodes may appear to occur independent of life stress. The kindling model is supported by multiple studies that found major SLEs occurring particularly in the year before the first episode of mood disturbance [114, 115, 121] or early on in the course of illness [122]. However, Bender and Alloy [114] found that many of these studies were methodologically flawed and offer only limited support to the widely cited kindling hypothesis.

The BAS dysregulation model is based on research showing that behavior is regulated by goals and rewards (when faced with goal-related cues) and a behavioral inhibition system (BIS) that triggers avoidance when a person is faced with cues related to threat or punishment [114, 120]. There is some evidence that in persons with BD, the BAS may be hyper-sensitive such that goal-related cues may trigger hypomanic behavior, while threat-related cues may trigger depression [114, 120]. 

The SRD model of diathesis stress is supported by several studies finding that SLEs, in combination with genetic differences, predict manic and depressive symptom recurrence [123] and delay in functional recovery [124] over the course of illness. On the side of genetics, Hosang et al. [125] found that for the worst depressive episodes in BD, stressful life events (SLEs) were significantly moderated by BDNF genotype—Val^66^Met polymorphism. On the side of environmental stress, diminished perceived social support and psychosocial stress appear to be particularly predictive of mood instability in BD [123, 126–128]. In this regard, there is evidence that SRD and disruption to the attainment of psychosocial goals are associated with the number of reported manic episodes [121, 129]. In addition, social rhythm irregularity predicts time to affective relapse [121], and there is some evidence that persons diagnosed with BD experience higher numbers of SLEs and greater SRD than people without psychiatric illness [130]. Taken together, these studies point to the possible development of a reciprocal loop between SRD and mood symptoms, in which SRD aggravates the genetic propensity toward mood disturbance, and mood symptoms in turn exacerbate SRD. 

In sum, these findings suggest that genetics carry an important influence on illness severity in BD, that SRD is a particularly destabilizing source of stress for people with a genetic predisposition toward BD, and that people with BD experience more psychosocial stress than people without mental illness. These factors may be central to understanding functional decline in BD. SRD alone, by definition, disrupts psychosocial functioning. Its effects in BD, however, might be compounded by the association between SRD and the recurrence of genetically triggered mood instability, which imposes a powerful and direct impediment to psychosocial development. 

## 7. The Integrated Model

**Figure 1:**
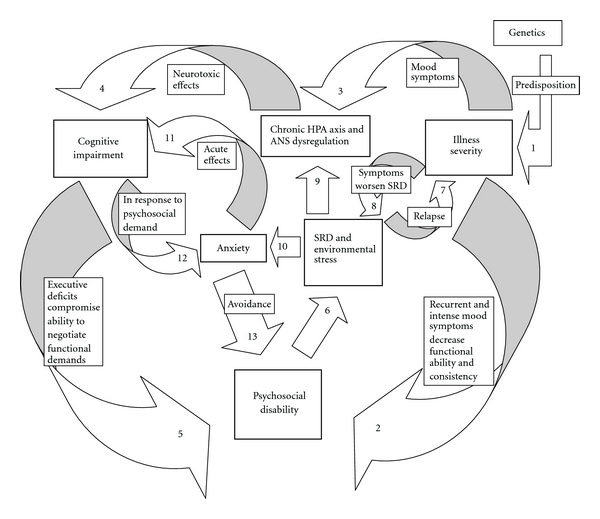
The malignant cycle in bipolar disorder.

Previous research illuminated many aspects of illness progression in BD, including factors that contribute to morbidity and psychosocial disability. This paper examines the effects of illness severity, cognitive impairment, anxiety, genetics, and psychosocial stress on functional outcome in BD. The interplay among these factors may be complex and involve reciprocal pathways.  Figure 1 presents 13 possible interconnected pathways that potentially trap people with BD in a malignant cycle that accelerates psychosocial decline. The numbers that appear on the arrows in the figure match those of the pathways described below.

**Pathway 1**

There is a strong genetic component in BD that influences the onset, severity, and progression of the illness.

**Pathway 2**

The symptoms of BD have a direct impact on psychosocial functioning. Recurrent mood disturbance, lingering residual symptoms between episodes, hospitalizations, comorbid substance use disorders, and psychosis disrupt the consistency of psychosocial engagement required for functional development.

**Pathway 3**

Recurrent episodes of mood disturbance result in chronic physiological stress related to the hyperarousal of the autonomic nervous system and HPA axis.

**Pathway 4**

The physiological effects of stress are neurotoxic and lead to cognitive decline over time.

**Pathway 5**

Cognitive impairment in general, and executive dysfunction in particular, hampers the ability to meet psychosocial demand.

**Pathway 6**

The difficulty in meeting psychosocial demand creates disruption to social rhythm and increases environmental stress.

**Pathway 7**

Environmental stress in general, and psychosocial stress in particular, aggravates the phenotypic expression of mood disturbance, leading to a more severe course of illness.

**Pathway 8**

The consequent intensification in symptoms and their recurrence exacerbate the disruption to social rhythm and environmental stress.

**Pathway 9**

Psychosocial stress contributes to chronic hyperarousal of the autonomic nervous system and HPA axis.

**Pathway 10**

Repeated experiences of psychosocial failure intensify anxiety related to psychosocial demand.

**Pathway 11**

Anxiety has acute effects on cognitive functioning during psychosocial challenges. Superimposed on cognitive impairment, anxiety further compromises attentional control and executive functions.

**Pathway 12**

The specific encounter between cognitive impairment and challenges in a psychosocial context worsens anxiety.

**Pathway 13**

The anxiety associated with functional challenges leads to avoidance of psychosocial demand and marginal psychosocial engagement.

## 8. Implications for Care

The model presented in Figure 1 approaches BD from a holistic perspective. Well-embedded in diathesis-stress notions, the model traces the roots of pathology and psychosocial dysfunction in BD primarily to the interaction between the person and the environment. The model places particular importance on the psychosocial environment, as opposed to other sources of stress that can aggravate the illness. Central to this notion is the goodness of fit between the person and the psychosocial environment. Chronic dissonance in this relationship may lead to a more severe course of illness and a malignant decline in functioning. Within any given individual, genetic predisposition toward BD remains constant; therefore, improvement may occur as a function of changes in the psychosocial environment. This conclusion may deserve particular attention when failure to thrive continues despite substantial therapeutic and pharmacological efforts to overcome the effects of the illness. In many such cases, psychosocial avoidance may lead to disability even in the absence of acute symptoms. In other cases, the misfit between the person and the psychosocial environment may override the effects of medication, so the person remains disabled by the recurrence of symptoms. 

In the current social and economic climate, goodness of fit between the person and psychosocial environment receives far less attention than pharmacological interventions. In BD, the beneficial effects of medications are powerful for many people, but they still offer limited remedy for the illness. Psychosocial disability in BD often lingers despite medication, possibly in part because medications typically do not alleviate cognitive impairment [131, 132] and may, in fact, aggravate it [133, 134]. Although medication can improve psychosocial functioning in BD in general by ameliorating affective symptoms [135], pharmacological interventions alone may not have sufficient power to overcome the destabilizing effects of psychosocial demands that exceed the person's functional capacities [7]. 

People with BD who strive to flourish against a current of psychosocial demand that is too stressful for their genetic level of stress tolerance may ultimately experience exhaustion, intense anxiety, and decompensation [7, 136]. Hospitalization may help to temporarily alleviate this experience. In this process, the chemical effects of pharmacology often reduce mood symptoms within the custodial environment of inpatient care. However, discharging the person into the same unworkable situation may result in recurrent decompensation and a sizable increase in the number and dose of prescribed medications over time. 

To remain stable under these circumstances, many people with BD may choose to disengage from the natural pursuit of psychosocial development and seek the protective benefits of disability. Initially, this may bring some relief; however, trading psychiatric symptoms for psychosocial disability may become problematic over time. The stagnancy and social marginalization that may be created by disability can be detrimental to a person's identity and self-esteem [66, 137–139]. As time passes, the developmental gaps from the person's cohort widen, and the consequent changes to the person's identity and belief system diminish the probability of reversing the trend from psychosocial decline to growth [140].

Aside from medication, psychosocial interventions and support groups are also vital to improving functional outcome in BD. Support groups and psychotherapy offer a context in which people can experience acceptance, appreciation and meaningful interpersonal connections. Some interventions such as interpersonal and social rhythm therapy (IPSRT) may also enhance psychosocial competence in BD [141].

At the same time, these efforts may not be powerful enough to override a misfit between genetic vulnerability to stress and psychosocial demand. If people are unable to maintain consistent social and professional growth that is commensurate with their potential outside therapeutic settings, their lives remain limited by psychiatric illness and functional disability. 

To overcome this problem, clinicians working with BD may need to develop expertise in helping people identify psychosocial contexts that facilitate growth. Learning to conduct, or at least interpret, cognitive assessments with ecologically valid interpretations would likely be fundamental to this process [142]. Clinicians who understand the interplay between a given profile of cognitive deficits and particular environments may be able to guide people toward settings that increase the likelihood of psychosocial success. Clinicians may also be able to provide persons with BD ongoing guidance with respect to goal-related expectations, pace of progress, and workload [143, 144]. 

Moving from assessment to implementing recommendations regarding psychosocial adjustment for persons with BD will require clinicians to pay particular attention to the anxiety related to psychosocial demand. The fear of repeated psychosocial failure can lead to the avoidance of functional challenges and to feelings of helplessness and hopelessness. Helping people with cognitive impairment and mood instability overcome the impediments these factors create may require a great deal of expertise and potentially even more highly specialized programs—for instance, an intervention may combine elements of IPSRT with vocational counseling tailored to BD [145], cognitive remediation [146], and other interpersonal therapy. Traditional practices of vocational counseling alone may not suffice. 

The delivery of psychosocial interventions aimed at improving social and occupational outcomes needs to be particularly sensitive to cognitive impairment and residual symptoms. As previously noted, longitudinal studies show that two key predictors of future social and occupational functioning in BD are subsyndromal depressive symptoms and cognitive deficits, particularly in executive functioning [25, 31, 147]. Cognitive impairment and residual depressive symptoms in BD have also been found to correlate with each other, independent of other outcomes [148]. Manic and depressive residual symptoms that are present during early remission from a mood episode also predict relapse [149], while cognitive dysfunction impedes the effectiveness of psychosocial interventions designed to improve functioning [145, 150] and reduces treatment adherence [151].

Given the impact of both residual symptoms and cognitive impairment on functioning, and the correlation between them, a thorough assessment of each should be included as part of the standard of care in BD. With respect to cognitive functioning, patient reports may not provide a sufficient indication of cognitive status, as these show weak correlations with objective assessments [152]. To adequately identify cognitive dysfunction in BD, assessment using a standard neuropsychological battery may need to become routine, as cognitive deficits in BD typically do not present when evaluated with the minimental status exam (MMSE) [55] commonly used by clinicians.

In the future, identified cognitive deficits may be addressed to some degree with direct interventions including compensatory [146] and restorative cognitive remediation programs [153] both manualized [154] and computerized [155]. Meta-analyses have found small-to-medium effect sizes for improving cognition using restorative cognitive remediation programs in several psychiatric conditions including schizophrenia [153] and substance use disorders [155]. In BD, findings so far are limited to a small, uncontrolled study involving a compensatory cognitive skills training program [146]. This study found that traditional CBT aimed at reducing residual depressive symptoms, combined with sessions teaching compensatory cognitive skills, resulted in significant improvement in occupational outcomes for eighteen people with BD. Several clinical trials aimed at determining the efficiency and occupational outcomes of restorative cognitive remediation and pharmacological interventions in BD are ongoing [156, 157] and await conclusion.

Even when not directly targeted, cognitive deficits in BD may require that psychosocial interventions such as psychoeducation be delivered in a highly structured manner that accommodates cognitive disability [145, 158, 159]. Multiple findings indicate that psychoeducational interventions aimed at relapse prevention are effective and may improve functioning in BD, highlighting the need for these interventions to be accessible to the cognitively impaired [159]. Finally, psychoeducation regarding cognitive impairment itself may help persons with BD learn supportive techniques and strategies to compensate for such deficits in occupational settings [146].

After clients take action to re-engage in occupational pursuits, counselors may need to help them persevere in the face of the natural frustrations that accompany efforts to obtain psychosocial accomplishments on an alternative schedule. Counselors may also need to assess and monitor the person's stress effectively. Taking significant steps toward psychosocial development in BD is desirable but can increase stress, and thus lead to relapse. Clinicians will likely be challenged to help clients manage the stress without abandoning their quest for psychosocial growth or resigning themselves to a state of disability. 

Given all of these challenges, progress toward psychosocial growth in BD may well be inconsistent. In many cases, a successful outcome of counseling would be to keep the growth from being eliminated completely in the face of recurring symptoms. Ultimately, a positive trend in psychosocial growth may be more important than measuring any one sizable change in outcome. Mild but valued movement toward growth with manageable stress may prove to be an effective mood stabilizer. Conversely, the absence of psychosocial growth may lead to a malignant decline in functioning. 

Finally, further advances in functional outcomes for persons with BD will probably require changes in the social climate. At present, few mainstream environments accommodate the special needs of people with BD. Moreover, stigma and discrimination against people with mental illness in the workplace remain major obstacles for psychosocial growth in BD [66, 68, 137, 160, 161]. Consequently, in most settings, the intensity of functional demands and inhospitable atmosphere may be too stressful to negotiate with sufficient long-term consistency. In the absence of ongoing support, the chronic mismatch between the functional limitations of persons with BD and the environmental demands they face greatly impedes their psychosocial adjustment and development. Developing effective support for psychiatric disability in mainstream settings may, therefore, improve clinical and functional outcomes. More broadly, mainstream support and an inclusive shift in social climate may be essential for curbing the downward psychosocial spiral that so many people with BD experience after illness onset. 

In conclusion, the factors that contribute to psychosocial impairment in BD may be looped together in intricate ways, creating an effect that traps people in a course of functional decline. Altering this downward trajectory may require both searching for and actively creating psychosocial environments that are hospitable to the specific needs of people who suffer from BD. In basic conception, psychosocial disability is environmentally dependent and not a constant. For this reason, in addition to medications and conventional forms of therapy, a strategic approach that enhances the goodness of fit between persons with BD and their psychosocial environment may change their possible functional outcomes. Most importantly, improving this fit may shift the lifelong path of persons with BD from malignant psychosocial decline to growth.
